# “it’s a medical condition … you need to support as much as possible”: a qualitative analysis of teachers’ experiences of chronic fatigue syndrome / myalgic encephalomyelitis (CFS/ME)

**DOI:** 10.1186/s12887-020-02461-7

**Published:** 2021-01-04

**Authors:** Amberly Brigden, Alison Shaw, Esther Crawley

**Affiliations:** Population Health Sciences, Bristol Medical School, University of Bristol, 1-5 Whiteladies Road, Bristol, BS8 1NU UK

**Keywords:** CFS/ME, Myalgic encephalomyelitis, Chronic fatigue syndrome, Paediatrics, Qualitative research methods, Primary schools, Teachers

## Abstract

**Background:**

An increasing number of children with complex health needs are being educated in mainstream classes. CFS/ME is a complex and disabling condition, and there is little guidance on how primary school teachers can support younger children with this condition. To improve care, it is important to understand what these children need in the school setting, and the barriers and facilitators to teachers providing this support. The aims for this qualitative study were to explore teachers’ views about CFS/ME, their experiences of supporting a pupil with CFS/ME and their perspectives on the barriers and facilitators to providing support.

**Methods:**

We recruited families from an NHS specialist paediatric CFS/ME service and families were eligible if the child was aged between 5 and 11 years and had a diagnosis of CFS/ME. We gained written consent/assent from families to invite the child’s teacher to participate in a qualitative interview. We contacted these teachers, gained written consent and then carried out semi-structured qualitative interviews. Interviews were audio-recorded, transcribed, anonymised and analysed thematically. Interviews took place between July 2018 and December 2018.

**Results:**

We interviewed 11 teachers; their pupil’s age ranged from 5 to 11 years and school attendance ranged from 0 to 80%. Theme 1: Most teachers provided rich descriptions of their pupil’s CFS/ME; they consistently described cognitive dysfunction and significant fatigue, but beyond this the symptoms varied from one account to the next (from mobility problems, to aches and pains, digestive problems, headaches, nausea and hypersensitivity). These teachers noted the ripple effects on their pupil’s social, emotional and academic functioning. Two of the eleven teachers said that they did not observe symptoms of CFS/ME, expressing a degree of scepticism about the diagnosis. Theme 2: Teachers described a close relationship with their pupil. They said they understood the individual needs of the child and portrayed positive and proactive attitudes towards providing support. The type of support provided included facilitating rest breaks and limiting strenuous activities; using practical strategies to address cognitive, physical, social and emotional difficulties; maintaining a connection with the child during their absences from school; and encouraging the child to talk about their health and wellbeing. Teachers noted that receiving formal confirmation of the child’s diagnosis enabled them to put this support in place. Theme 3: The adaptations they described were often intuitive, rather than being based on a knowledge of CFS/ME. Teachers wanted more resources to increase their understanding of the condition and its management.

**Conclusions:**

Primary school teachers want to provide effective support for children with CFS/ME. Clinical services should consider working in collaboration with teachers to equip them with evidence-based strategies for CFS/ME management in the primary school setting.

**Supplementary Information:**

The online version contains supplementary material available at 10.1186/s12887-020-02461-7.

## Background

Between 13 and 27% of children are affected by one or more chronic health conditions [1], and the prevalence is increasing [2, 3]. Chronic health conditions affect many aspects of the child’s life, with consequences that endure into adulthood [4]. Chronic health conditions can be managed with evidence-based behavioural interventions. Examples include incontinence interventions which promote adaptive drinking and voiding behaviours (Urotherapy) [5], activity management interventions for paediatric CFS/ME [6], and diabetes interventions which focus on promoting blood glucose monitoring, selection of healthy food choices, and adherence to insulin therapy or other medications [7]. Behavioural interventions have the potential to improve symptom management, reduce physical disability and improve emotional and social functioning [8–10].

Younger, primary school-aged children (5–11-years) typically do not have the capacity to manage their treatment plans independently. They depend on the adults around them for support [11], and as children spend a significant amount of time at school, this includes reliance on their teacher. In the UK, an increasing number of children with chronic health conditions are being educated in mainstream classrooms, and school are mandated to provide support. UK education policy promotes inclusive education, the equality act (2010) states that reasonable adjustments must be made to alleviate disadvantage, and section 100 of the Children and Families Act (2014) places a duty on governing bodies to make arrangements to support children with medical needs. Despite this, these children are often not supported adequately at school [12].

Managing a chronic health condition is a significant responsibility for teachers, and they face barriers in this role. This includes gaps in their knowledge, concerns about the risk and responsibility of managing a condition, large class sizes, the burden of the curriculum, a lack of training and resources, and a lack of inter-agency communication [13, 14]. For each health problem the teacher encounters, they need condition-specific knowledge and skills [15]. Therefore, for any particular condition, it is important to ascertain what information teachers require, what actions they can take to support the child and the barriers and facilitators to the teacher providing the necessary support. To understand this, it is essential to capture the views of school staff [16].

Paediatric CFS/ME is a complex condition that includes a range of symptoms such as debilitating fatigue, pain and nausea [17]. Approximately 0.1–2% of adolescents are affected by CFS/ME [18]. It is less common in primary school age (5–11 years), but the prevalence in this younger age-group is unknown. Primary school-aged children with CFS/ME are significantly disabled by the condition; with low levels of school attendance and high levels of fatigue, anxiety, physical disability and pain [19]. The National Institute for Health and Clinical Excellence (NICE) guidelines recommend cognitive and behavioural interventions which help with management of activity, sleep, symptoms and co-morbidities, such as mood disorders and pain. NICE guidelines state that schools should be involved in treatment [17], but they fail to provide details on how to implement this in practice, nor do they address the specific issues of working with primary schools. Research focusing on adolescent populations suggests that school staff should support paediatric CFS/ME by being informed about the disease, recognising symptoms in the student, educating other staff and families, and facilitating adaptations in the school environment and educational curriculum [20]. However, secondary schools are often confused about the nature, origin and cause of the illness [21], and often hold unrealistic expectations of what the child can do [22]. Health professionals and families report that schools vary in their attitudes and the support they provide to children with CFS/ME [22]. We are not aware of any research specifically focusing on CFS/ME in the primary school setting.

We do not know enough about the teacher’s experience of supporting a younger child with CFS/ME. The aims of this qualitative study were to explore teachers’ views about CFS/ME, their experiences of supporting a pupil with CFS/ME and their perspectives on the barriers and facilitators to providing support.

## Methods

### Design

We undertook qualitative interviews to gather the views of school staff who had experience of supporting a primary school-aged child (5–11-years) with CFS/ME.

### Participants and recruitment

We recruited teachers of children taking part in the EXPLORER study. The EXPLORER study was a longitudinal cohort study exploring the epidemiology of CFS/ME in younger children, with integrated qualitative methods to explore the lived experiences of stakeholders. We recruited families from a specialist NHS paediatric CFS/ME service. Families were eligible if the child had a confirmed diagnosis of CFS/ME [17] and was aged 5–11-years.

The qualitative element was initially designed to explore the experiences of families and clinicians. As the qualitative interviews progressed, we recognised the need to explore the views of school staff, and so we amended the protocol to allow us to interview teachers.

We provided families with a participant information sheet about the school qualitative study. This included information sheets designed for parents/carers, children aged 5–7 years and children aged 8–11 years. We explained that the school qualitative interviews were an optional element of the study. If families wished to take part, we gained written consent from parents/carers and written assent from children aged 8–11 years. For children aged 5–7 years, we did not take formal written assent, [23, 24] but we involved children in recruitment discussions, checked for a clear signal that they were willing to take part and used an “ethical radar” [25], to attend to verbal and behavioural signs that the child did not wish to participate. The participant information sheets and consent/assent processes were developed in consultation with a young person advisory group. Fifty-six percent of the families (*n* = 28/50) consented to the school qualitative study.

After gaining consent from families, we contacted the school that was detailed in the participant’s clinical notes (information provided by the family at their initial assessment with the clinical service). We sent the school a participant information sheet, and we followed this up with a telephone call to offer further information and answer questions. We did not ask the family to specify a point of contact within the school, and therefore we worked with the school to identify the staff member who would participate in the study (typically the child’s class teacher). If school staff wished to take part, we arranged a meeting (either at the school or at the university), and we obtained full written consent. School staff could have any professional role, but to be eligible, they had to have direct experience of supporting the pupil with CFS/ME. The interviewer clarified that their role was that of a researcher, and not a clinician. They made teachers aware that they would not disclose any personal/ clinical information about the pupil, and the teacher was advised to contact the clinical service should they wish to have a clinical discussion about the pupil.

### Procedure

The lead author (AB) conducted one-to-one face-to-face interviews using a semi-structured topic guide [26] (see Additional file 1 for the topic guide). The topic guide was based on the literature on the management of chronic health conditions within schools, our previous qualitative work with young children with CFS/ME and our research aims. The topic guide included open questions with prompts, designed to explore: participants’ knowledge of CFS/ME, how the pupil with CFS/ME presented in the classroom, how the teacher approached the management of CFS/ME, facilitators and barriers to providing support, and issues surrounding communication with the specialist CFS/ME service. Participants were invited to add anything not covered by the topic guide, and were encouraged to talk for as long as they needed. The topic guide was iterative, and we amended these as the interviews progressed. For example, we noted that during the interviews, teachers tended to give a positive presentation of the teacher’s role in supporting the child with CFS/ME. We wanted to “get below the surface” [26], and explore both the positives and the challenges. Thus, in a second iteration of the topic guide, we included more prompts to explicitly ask about the challenges, framed in a non-judgmental way to put teachers at ease. We audio-recorded interviews using an encrypted digital recorder. Audio recordings were then transcribed verbatim and anonymised.

We used purposive sampling [27], maintaining a recruitment framework to ensure diversity in the pupils’ ages and their self-reported levels of school attendance. We considered sample size throughout data collection and analysis. We ceased recruitment when we believed new interviews were no longer adding additional insights for the aims of the analysis (pragmatic saturation) [28], with the acknowledgment that qualitative data is rich and complex and that there is always potential for additional interviews to add additional insight [28]. We also considered “information power” [29]; whether the sample was sufficiently diverse and the data of sufficient quality to answer the research questions.

### Analysis

We imported transcripts into the qualitative data management software Nvivo [30]. We used thematic analysis, informed by the stages proposed by Braun & Clarke [31]. Analysis was ongoing and iterative, commencing soon after each interview, with early analysis shaping the data gathered in subsequent interviews. The lead author (AB) analysed all transcripts. To begin, each transcript was reviewed in its entirety to gain familiarity with the interview as a whole. This was followed by systematic line-by-line coding of the transcript, assigning descriptive codes. Our codes were primarily inductive and data driven. We reviewed the codes across the transcripts and grouped these into broader thematic categories. As analysis progressed, we considered new interviews in the context of the existing codes and themes, and we revised and refined the themes to account for the new data. We developed themes to reflect the complexity in the data and to represent both common and divergent views. An experienced qualitative researcher (AS) independently conducted preliminary coding of a subset of the data, met with AB to agree on the coding framework, and contributed to the interpretation and write-up of the final set of themes.

## Results

### Participant characteristics

We interviewed 11 school personnel, all of whom were female. 64% of participants were the pupils class teacher, 36% had a senior/ leadership position in the school (e.g. head, deputy head, head of year), one participant (9%) was a SENCO and one (9%) was an intervention officer responsible for managing safeguarding and pastoral issues (the total of these percentages exceeds 100% as some participants had dual roles within the school). The index pupil’s ages ranged from 5 to 11 years and 64% were female. Table 1 presents the characteristics of the school, school staff and the index pupil. One teacher was interviewed at the university, the rest were interviewed at their school premises.

**Table 1:** Characteristics of the school, school staff and index pupil

ID	Pupil’s age	Pupil’s school attendance (%)	Type of school	Participant’s relationship to pupil
1	KS^a^2	60	State^b^ school	Class teacher
2	KS1	80	State school	Class teacher
3	KS1	20	State school	Class teacher
4	KS1	20	State school	Intervention officer responsible for managing safeguarding & pastoral issues
5	KS2	60	State school	Class teacher
6	KS1	80	State school	Class teacher
7	KS1	80	State school	Deputy head & class teacher
8	KS2	40	State school	Senior lead teacher & class teacher
9	KS2	20	State school	Head of year
10	KS2	0	Private school	Head of year
11	KS2	0	Private school	Specialist Educational Needs Co-ordinator (SENCO)

^a^ Key Stage (KS) 1 = 5–7 years; KS2 = 8–11 years

^b^ State school = government funded schools

As most participants were teachers, we use the term teacher throughout the results.

### Themes

Theme one captures the teacher’s rich descriptions of their pupil’s CFS/ME; the range of symptoms they noted, and the effect on their pupil’s social, emotional and academic functioning. It also presents alternative perspectives from teachers who were more sceptical of the diagnosis. Theme two presents the proactive and positive attitudes that teachers portrayed toward supporting their pupil, and describes the types of support offered. Theme three discusses how teachers made intutive responses, rather than being based on a knowledge of CFS/ME. This theme presents teachers views on optimal ways to increase their understanding of the condition.

#### Theme 1: the physical symptoms of CFS/ME and the ripple effect on social, emotional and academic functioning

All but two of the teachers described the visible and disabling effects of CFS/ME. Teachers consistently observed cognitive difficulties, using terms such as “*foggy head*” (ID4). They noted a “*slow*” (ID8) pace of work; problems with multi-tasking (“*he could only handle one thing at a time*”, ID9); the pupil becoming “*overwhelmed*” (ID3) by sensory information (loud, busy classrooms and apparatus); and problems with memory and concentration. Teachers also consistently noted debilitating fatigue. Beyond this, different teachers described different types of symptoms, including decreased mobility, pain (“*aches all over her body*”, ID5), nausea, digestive problems, hypersensitivity, headaches and a pale complexion.

*was absolutely exhausted … the colour drained from his face, he moved very slowly, he doesn’t travel very long distances … he’s come in and hasn’t been able to say a word he’s been extremely tired* (ID9).

Teachers explained that the illness had a ripple effect on the pupil’s social, emotional and academic functioning. Teachers emphasised the impact of CFS/ME on peer relationships. In some cases, classmates behaved in ways that stigmatised the child and restrictions on activity and absences could result in the child’s “i*solation*” (ID10).

*she has quite big bags under her eyes and a lot of them think she’s ill … children who aren’t as polite, have said things like, yeah ‘she looks like a ghost’ or ‘I don’t want to go near her because I’ll catch it’ and ‘she’s got a disease’ things like that.* (ID3).

*not being able to go out at playtime, I think that really affected her and her friendships* (ID5).

Some teachers noted the emotional impact on their pupil, reporting that their pupil was “*very emotional*” (ID5), “*she’ll go really low and really angry*” (ID6). Teachers described general anxieties, school-specific anxiety, low self-esteem, negative cognitions, and a sense of the child getting “*lost in the diagnosis*” (ID3).

Though the majority of teachers observed symptoms of CFS/ME, two teachers said they did not observe any symptoms and expressed scepticism about the diagnosis: “*he’s not presenting as a child who is ill*” (ID11), “*the tiredness, I mean she didn’t ever seem that that was a big problem* … *she seemed to function quite well in the classroom*” (ID2). Another teacher observed symptoms (“*tummy ache … a headache*” ID10) but wondered whether this was “*anxiety related … stress related*”(ID10) and questioned if these psychological elements were a “*consequence or a cause*” of her physical symptoms.

#### Theme 2: close relationships and tailoring support to the individual needs of the child

Teachers explained that receiving confirmation of the diagnosis from clinic was an important enabler to them providing support.

*we had the letter with the formal diagnosis on it …* [the school] *agreed without hesitation we had the back-up letter from the consultant so it all went really smoothly* (ID8).

Once they received this, they were proactive in implementing a diverse range of adaptations to support the individual physical, emotional, social and academic needs of their pupil.

*it’s a medical condition, the child is not well … You need to support as much as possible* (ID1).

They explained that they were in a good position to offer this support due to their close and consistent relationships with their pupil (“*he’s with me pretty much all day, every day*”, ID1, “[I] *get to know each individual child*”). This theme describes the different types of support that teachers offered.

##### Managing the child’s activity

Teachers understood that a key element of CFS/ME management was regulating the child’s activity to avoid overexertion. Teachers worked “*together*” with families to negotiate a reduced timetable for the child. They based this on the child’s individual academic needs, subject preferences, social needs, the times of day when the child had the most energy and lessons which were more strenuous.

*I worked with mum basically to look at the timetable, look at where he could miss school and not have too much of a detriment to his learning … so we looked at his strengths and weaknesses in terms of his literacy and his maths and other subjects– together we co-ordinated a timetable*. (ID1).

Teachers facilitated breaks for their pupil in lessons and breaktimes, during which children were encouraged to rest and engage in low energy activities (“*to relax and lie down*”, ID5). Teachers provided quiet and comfortable spaces, providing items such as “*cushions*” (ID2) “*beanbags”* (ID3), “*blanket … some comfort things*” (ID5). Teachers prompted the child to take planned breaks, as well as being vigilant to signs of fatigue and recommending impromptu breaks as and when needed:

*I know to look if he’s looking tired … I can sort of say are you okay, do you need some time, do you want to go and sit in the library* (ID1).

Instead of focusing on restriction and limitation, teachers reframed these breaks as meaningful roles or enjoyable activities. Examples included supervising younger children, helping at the school’s reception desk at break times and being a conductor in music lessons.

*I used to say to the music teacher she can’t stand and play the violin … she can’t actually physically do that, but she could possibly conduct them to keep them all in rhythm* (ID8).

##### Responding flexibly to cognitive, physical and emotional difficulties

Teachers made adaptations to cater for cognitive difficulties. They simplified tasks and provided extra time *(“take as long as you want over it*”, ID1). To support the child with writing, teachers scribed, provided iPads, and suggested alternative activities to writing. Teachers also devised bespoke lessons plans to cater for cognitive issues and absences.

*made a tailored curriculum … I would plan all of this and I would have the long-term plan for her for the months that were left in school … the medium term work that week and then I would make the photocopies and everything I needed for her* (ID10).

Teachers also responded flexibly to individual needs: they catered for eating difficulties by allowing food and drink in the class and prompting the child to eat little and often. They supported the management of pain through behavioural techniques and medication. For those with mobility problems, they limited the distances the child had to travel in the school environment.

*we couldn’t put him in a lesson on one side of the school to the other … … I limit the sort of distance that he has to travel if you like* (ID9).

Teachers employed strategies to manage the emotional needs of their pupil such as challenging negative thinking, building self-esteem, encouraging relaxation and mindfulness and “*talking about emotions*” (ID6).

*I tried relaxation … mindfulness, we found that really helped, like the meditation I found like a couple of videos so she’d take an iPad out into a quiet space … she’d come back in like, “Yeah, it did. It did work”, cos it like talks to her and she has to concentrate on her breathing* (ID5).

##### Keeping a connection with school life

Teachers felt that it was important to try and make children feel “*as included as much as possible*” (ID7) and feel “*present even though absent*” (ID10). There was wide variation in how schools tried to maintain engagement in school life, including sending work home, organising contact from classmates, using technology (“*Skype or webcam so [child] realises that she’s still part of this school*”, ID3), home-schooling and hospital education.

##### Encouraging communication about health

Most teachers reported that their pupil “*wasn’t good at verbalising*” (ID8) their symptoms, feelings, needs and preferences. All teachers felt that it was important to try and help communication. Some described tools/ techniques to facilitate this, such as “*show a card*” (ID5), symbolising their feelings and needs, and “*a check-in system*” (ID7), a set time for the child to communicate their feelings.

##### Facilitating peer support

Teacher described their role as helping the child to maintain friendships. They organised contact at a manageable level to the child (“*we got him together with just four friends, now half his tutor group wanted to see him which would … that it would have been far too overwhelming”* ID9), ensured that the child had company on rest breaks, and arranged for peers to send things to the pupils home.

*they sent him emails, they sent him video clips, they sent him photos of things they did* (ID11).

Most teachers felt that it was important to talk to the class to raise “*awareness*” (ID6) and to promote supportive and empathetic attitudes.

*children are going from a place of ignorance, to a place of ah, I get it now. Often a lot of picking on children issues disappear when they’ve had that peer awareness work* (ID1).

They indicated that resources to help them talk to their class about it would be “*be really helpful*” (ID5). Teachers suggested “*PowerPoint*” (ID 1,2,3,8) “*class activities*” “*pictures*” (ID2) a “*child-friendly way explaining it*” (ID2), “*fun*” explanations, “*some like basic child-friendly information*” (ID6). They indicated that these were available and frequently used for other conditions/difficulties such as ADHD, Downs Syndrome, ASD, Dyslexia, hidden disabilities, Diabetes, bereavement, epilepsy, obesity and visual impairment.

Teachers described barriers to supporting the individual needs of the child while managing large classes. They talked about the additional time, effort and staffing that was needed to support some of the adaptations. Further, “*unpredictable*” attendance meant teachers were unable to plan and were forced to make “*off the cuff*” (ID8) lessons plans for the child. However, on the whole, teachers viewed adaptations such as reduced timetable as feasible and acceptable, and they worked flexibly to facilitate these.

Theme one described three teachers who said they did not observe the symptoms or who questioned the diagnosis. In terms of the support these teachers offered, two said they worked to “*maintain contact*” (ID11) during the child’s absences and developed tailored academic programmes to make up for missed schoolwork. They stated that the child did not require any other adaptations for their health (“*nothing of that has ever been needed*” ID10). The third made adaptations at the family’s requests, such as facilitating rest breaks and reducing timetables.

#### Theme 3: proactive in providing support but lacking formal knowledge of the condition

Three teachers had prior knowledge of CFS/ME due to personal experiences, knowing a family or friends who had the condition. The rest stated that they had “*very little*” (ID2) prior knowledge and received little information or guidance from the clinical team. This meant that the adaptations the teachers made for their pupil with CFS/ME were based on intuition (“*I basically made it up as I went along”,* ID5), their educational expertise (“*I kind of just used my own experience”*, ID8) and from conversations with the child’s parents/carers. As detailed in theme two, this did lead to proactive responses and individualised care plans for the child. However, in some cases, teachers felt concerned that they were making the right decision, and there were examples of strategies that were inconsistent with evidence-based treatment, for example, encouraging the child to take naps.

*we just kind of did everything that we could but didn’t know if it was the right thing or the wrong thing either really, I think that was the scariest part* (ID5).

Teachers said there was a lack of information and wanted better resources about the condition and how to manage it in the classroom in the form of leaflets and training. They contrasted the limited information with the helpful resources available for other medical conditions (allergies/ EpiPens, asthma, diabetes, ADHD, ASD, Crohn’s, epilepsy, dyslexia dyspraxia and the learning conditions). Teachers described the diverse ways that information was provided for these other conditions, from awareness-raising campaigns (“*an autistic day and a diabetes day and an EpiPen afternoon”,* ID3), in-house training from school nurses and SENCOs (“*a talk either from the school nurse or the first aid lead about diabetes, epilepsy … Crohn’s and diabetes*”, ID4) and face-face and online external training from the local authority and from NHS services. Some teachers felt they needed more intensive, “*direct conversation”* (ID4) with clinicians to gather individualised information about the child.

## Discussion

### Summary of findings

This is the first study to explore the views of teachers who had experience of supporting a younger child (5–11-years) with CFS/ME. Most teachers described CFS/ME as a disabling condition. These teachers consistently described cognitive dysfunction and significant fatigue, but beyond this, the symptoms varied from one account to the next (from mobility problems, to aches and pains, digestive problems, headaches, nausea and hypersensitivity). Two teachers did not observe symptoms of CFS/ME, expressing a degree of scepticism about the diagnosis. Teachers described a close relationship with their pupil. They said they understood the individual needs of the child and portrayed positive and proactive attitudes towards providing support. This included facilitating rest breaks and limiting strenuous activities; using practical strategies to address cognitive, physical, social and emotional difficulties; maintaining a connection with the child during their absences from school; and encouraging the child to talk about their health and wellbeing. Teachers noted that receiving formal confirmation of the child’s diagnosis enabled them to put support in place. Most teachers lacked prior knowledge of CFS/ME and felt there was limited information available. Because of this, the adaptations were often intuitive, rather than evidence-based.

### Strengths and limitations

We captured diversity with respect to the index pupil’s ages, gender and levels of school attendance. Schools were predominately state schools (82%, which approximately reflects the composition of state schools in the UK [32]) and we recruited schools from counties across the South-West of England, both rural and urban areas. The themes we developed capture the views of all participants, as we attend to divergent/negative cases [33].

Participants in qualitative interviews may provide socially desirable responses. We worked to gain a nuanced narrative by probing about the challenges of supporting a child with CFS/ME. Despite this, participants mainly portrayed positive attitudes. This may be an accurate reflection, or may be a result of participants self-censoring negative views. We also recognise that teachers were a self-selecting sample, and those who declined/did not respond may hold more negative views. All of the participants were female, and the challenges in recruiting male participants is likely due to the fact that approximately 85% of primary school teachers are female [34]. There may be gender differences in teachers views and experiences of CFS/ME, and we may not be able to generalise our results to male primary school teachers.

### Implications in the context of the literature

Teachers described proactive responses and expressed a sense of responsibility toward their pupil with CFS/ME. This is consistent with studies of other chronic health condition, which report that teachers feel central in supporting their pupil’s additional academic needs, emotional wellbeing, the social consequences of illness, and physical symptoms [13]. However, the positive attitudes in this study are somewhat surprising, given the history of CFS/ME. It has been a stigmatised condition [35], particularly in younger children where there has been little research [19] and historical claims that diagnosing children is harmful [36]. Although this study may indicate a shift in attitudes, it also reveals that it is still overlooked, with the teachers describing a lack of parity with other health conditions.

Teachers responded intuitively to meet the individual needs of the child. Intuition is an important part of professional decision-making which arises from an accumulation of experience [37]. Teachers described using strategies that were consistent with evidence-based management approaches for CFS/ME and chronic health conditions more generally. Examples include reducing school timetables, facilitating rest breaks, encouraging relaxation strategies, reducing isolation and promoting social support [6, 38]. This indicates that there is potential for evidence-based approaches to be implemented in the classroom setting. However, teachers lacked a formal knowledge about CFS/ME and there were examples of strategies that were inconsistent with clinical recommendations for CFS/ME (e.g. encouraging the child to nap). This suggests that teachers existing expertise need to be supplemented with a formal knowledge of the condition and its management. They need more information and support, to employ principles of NICE recommended behavioural interventions (e.g. activity management and graded exercise therapy) in the class setting.

Clinical services may be positioned to provide this information. This idea is consistent with multisystemic models of care where families, schools and health care systems work together to support the child [39]. It may be beneficial for clinics to focus on providing information that is pertinent to the teacher’s role: the variety of presentations they may encounter (symptoms of CFS/ME vary between individuals and fluctuate in intensity and severity [17]); managing physical, cognitive and emotional symptoms in the classroom; managing absenteeism; explaining the condition to peers and supporting the child with maintaining friendships. Individual health care plans [40] are designed to facilitate conversations between teachers, families and health service and are mandated by the Department of Education. None of the teachers reported using this tool, and this suggests a need to explore the barriers to using this tool and interventions to increase its use for CFS/ME.

Teachers talked about the potential for creative and technological solutions to help them manage absenteeism. This is consistent with tool available such as using toys to represent children in the classroom [41] and webcams to virtually extend the child’s participation and increase their social presence [12]. However, it is necessary to understand if these approaches are acceptable to younger children and their families.

## Conclusion

This is the first study to explore the views of teachers who had experience of supporting a younger child (5–11 years) with CFS/ME, and we have developed recommendations based on the findings of this study. More support and resources are needed for teachers supporting a younger child with CFS/ME. There are no evidence-based school interventions, and further research is required to develop and test them.

## Supplementary Information

**Additional file 1.**

## Data Availability

The datasets generated and/or analysed during the current study are not publicly available due to the nature of the data (i.e. interviews containing highly personal information) which precludes sharing this publicly to preserve participant confidentiality and anonymity.

## Abbreviations

CFS/ME: Chronic Fatigue Syndrome / Myalgic Encephalomyelitis
NICE: National Institute for Health and Clinical Excellence
NHS: National Health Service
KS: Key Stage
SENCO: Specialist Educational Needs Coordinator
