# Supplementary material for: “it’s a medical condition … you need to support as much as possible”: a qualitative analysis of teachers’ experiences of chronic fatigue syndrome / myalgic encephalomyelitis (CFS/ME)
Source: BMC Pediatr. 2021 Jan 4;21:6. doi: 10.1186/s12887-020-02461-7 (PMC7780629; doi:10.1186/s12887-020-02461-7)
Supplement: Supplementary file 1 — Additional file 1. [file 12887_2020_2461_MOESM1_ESM.docx]

**Topic Guide: Teacher**

This topic guide is for in-depth, semi-structured interviews with teachers to explore their experiences of supporting a pupil with CFS/ME.

**Prior to start of the interview**

- Check the participant has read the information sheet. Verbally go through information and answer any questions that the participant has
- Remind participants that interviewer is from the research team and not from the clinical team. Remind them that individual/ identifiable feedback from the school will not be relayed to the clinical team.
- Remind participants that interviewer cannot impart any clinical/other information about the child.
- Complete consent forms.
- With consent, turn on audio recorder

**Section 1: Contextual information**

- Briefly, can you talk to me about your role in the school and relationship to [pupil] (e.g. class teacher, head teacher etc).
- Can you talk to me about what you know about CFS/ME

**Section 2: Their pupil with CFS/ME**

Note: the facilitator might also ask additional questions to clarify information/ use probes encourage the interviewee to say more about a particular topic.

- Can you talk to me about your pupil with CFS/ME?

Prompting questions:

- Can you talk about how your pupil presents with CFS/ME (e.g. symptoms)?
- Can you talk about how the child copes with school?
- Can you talk about the impact of the condition on your pupil?

**Section 3: Supporting the child with CFS/ME**

Note: the facilitator might also ask additional questions to clarify information/ use probes encourage the interviewee to say more about a particular topic.

- Can you talk to me about you respond to the needs of your pupil?

Prompting questions

- Do they require any adaptations in the class/ school setting (e.g. Any adaptations relating to work, friendships, etc.) ?
- Can you talk about how you work with the child’s parent/carer?

**Section 4: Working with clinical services**

- Did you have any contact with the CFS/ME service? Can you talk to me about this?

Prompting questions

- How/ what did the clinic communicate? How regularly?
- What recommendations did they make- how did you respond to these?
- Did it seem like the right level of contact?
- What was helpful / what wasn’t helpful?
- How could things be improved?
- If the teacher did not have any contact/ recommendation from clinic. Explain: Clinicians may recommend some of the following for children with CFS/ME: reducing school timetables; missing PE lessons; not taking exams; taking rest breaks during school time; not going outside for play times.
- What do you think about this?
- How manageable is this for this school?
- What might be helpful for the school?

**Section 5: Close**

- Are there any issues that we have not talked about that you would like to raise?
- Clarify what happened next and who the participant can contact if they have any questions.
- Thank participants for their contribution.
